# AID and APOBECs as Multifaceted Intrinsic Virus-Restricting Factors: Emerging Concepts in the Light of COVID-19

**DOI:** 10.3389/fimmu.2021.690416

**Published:** 2021-07-01

**Authors:** Anastasia Meshcheryakova, Peter Pietschmann, Philip Zimmermann, Igor B. Rogozin, Diana Mechtcheriakova

**Affiliations:** ^1^ Department of Pathophysiology and Allergy Research, Center of Pathophysiology, Infectiology and Immunology, Medical University of Vienna, Vienna, Austria; ^2^ Nebion AG, Zürich, Switzerland; ^3^ National Center for Biotechnology Information, National Library of Medicine, National Institutes of Health, Bethesda, MD, United States

**Keywords:** AID, APOBECs, APOBEC4, AID/APOBECs gene expression signature, lymphoid structures, germinal center, SARS-CoV-2, COVID-19

## Abstract

The AID (activation-induced cytidine deaminase)/APOBEC (apolipoprotein B mRNA editing enzyme catalytic subunit) family with its multifaceted mode of action emerges as potent intrinsic host antiviral system that acts against a variety of DNA and RNA viruses including coronaviruses. All family members are cytosine-to-uracil deaminases that either have a profound role in driving a strong and specific humoral immune response (AID) or restricting the virus itself by a plethora of mechanisms (APOBECs). In this article, we highlight some of the key aspects apparently linking the AID/APOBECs and SARS-CoV-2. Among those is our discovery that *APOBEC4* shows high expression in cell types and anatomical parts targeted by SARS-CoV-2. Additional focus is given by us to the lymphoid structures and AID as the master regulator of germinal center reactions, which result in antibody production by plasma and memory B cells. We propose the dissection of the *AID*/*APOBEC*s gene signature towards decisive determinants of the patient-specific and/or the patient group-specific antiviral response. Finally, the patient-specific mapping of the AID/APOBEC polymorphisms should be considered in the light of COVID-19.

## Introduction

Maintaining the integrity of our genetic material is a prerequisite for proper long-term hierarchical functioning of cells, cell-based tissues, and tissue-based organs. This guarantees the full-fledged transfer of genetic material to the next generation and the maintenance of physiological, disease-free conditions. Multimodular systems monitor unwanted editing of genetic material, flag its presence and repair it (1–3). In parallel to preventing degeneration, circumstances exist in which DNA- and/or RNA-modifications must be reinforced to ensure effective protection. This applies to the mutagenic cellular factors of the AID (activation-induced cytidine deaminase)/APOBEC (apolipoprotein B mRNA editing enzyme catalytic subunit) family, composed in total of eleven members and evolutionally acting in both adaptive and innate immune responses (4–6).

**Figure 1:**
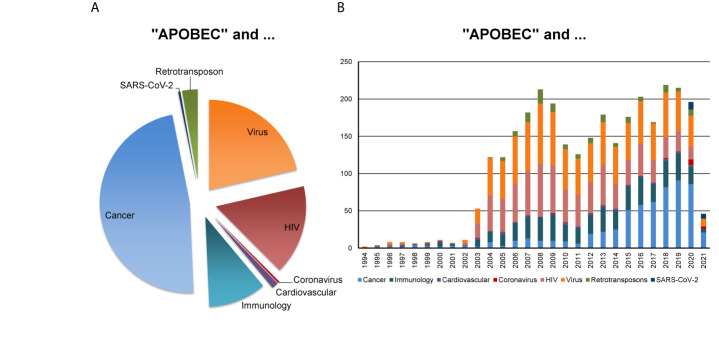
APOBEC-related research in various disciplines. PubMed-based search was performed using the combination of keywords “APOBEC” AND each of the eight indicated terms such as “Cancer”, “Immunology”, “Cardiovascular”, “Coronavirus”, “HIV” for human immunodeficiency virus, “Virus”, “Retrotransposon” (assessed on February 23, 2021). The outcome, as measured by the number of corresponding scientific publications, reveals **(A)** the proportion of scientific publications coming from indicated research areas and **(B)** the corresponding publications, published in the years between 1994 and 2021.

In this perspective, we bring up for discussion the strategic relevance of the AID/APOBECs for the understanding of the patient-specific nature of SARS-CoV-2 replication and viral adaptation, mutational landscape and pathobiology, and of the antiviral immune response. **Figure 1** reflects the major research areas related to the APOBEC family and the emerging interest of the scientific community for the role of APOBECs in the context of SARS-CoV-2.

The current knowledge on other aspects of AID/APOBEC biology including their origin, evolution, paralogs, structural features, cellular location, as well as the special role of AID/APOBECs in cancer mutagenesis, as well as lessons and models emerged from HIV-APOBECs counteractions can be found in excellent review articles (7–12).

## Intrinsic Antiviral Host Defense System Editing the Genetic Material of the Virus

The AID/APOBEC family represents the crucial part of the intrinsic host defense system that provides rapid and robust protection against endogenous retrotransposons and retroviruses as well as exogenous viruses (10, 13).

From the mode of action, the AID/APOBEC proteins are cytosine-to-uracil (C-to-U) deaminases, enzymes that physiologically target nucleic acids (9). An important point here is that the members of the AID/APOBEC family might have different preferences for either DNA or RNA as substrates (14–16). The latter underlines the potential relevance for restriction of viral RNA, counteracting the virus type which triggers some of the most persistent and complex challenges for public health and medicine.

Although solid data regarding nucleotide context specificity of AID/APOBEC enzymes, also known as mutable motifs, exist at the DNA level (17), our knowledge of mutable motifs and other context features associated with AID/APOBECs at the RNA level is scarce (14, 16, 18). Addressing mutable motifs represents a powerful approach to study molecular mechanisms of mutations linked to genetic diversity and adaptation of virus populations in the course of natural infection (17, 19).

APOBECs, with the best studied APOBEC3 subfamily comprising seven members (APOBEC3A, APOBEC3B, APOBEC3C, APOBEC3D, APOBEC3F, APOBEC3G, APOBEC3H), act against a wide variety of viruses including HIV-1 (RNA type), hepatitis B virus (DNA type), herpesviruses (DNA type), and parvoviruses (DNA type) (20). Recent discoveries demonstrated the APOBEC3-mediated restriction of viruses from the *Coronaviridae* family (15), including very first indications for the APOBEC-driven editing of RNA in SARS-CoV-2, a single-stranded positive sense RNA virus (16).

Consistent with the potential involvement of APOBECs in the process of coronavirus editing, several authors have identified an overrepresentation of C-to-U transitions in the SARS-CoV-2 genome. Specifically, the preponderance of C-to-U transitions was identified to occur within the sequence context targeted by APOBECs and could be attributed to structural configurations preferred by these antiviral proteins (8, 16, 21–23). Moreover, the C-to-U substitutions were shown to be associated with non-synonymous mutations, thus changing the amino acid sequence and, as potential consequence, resulting in a shift in the ratio of hydrophilic to hydrophobic protein areas (23, 24). This is of particular relevance for the masking or unmasking of the immunogenic epitopes of viral proteins, which in turn drive the specific immune responses ensuring the fight in the course of COVID-19. The arising question whether the APOBEC-driven shaping of the SARS-CoV-2 genome creates antigenic epitopes, which can be better seen by the cells of the adaptive immune system due to more stable presentation by antigen presenting cells in the context of MHC molecules and/or can ensure stronger binding to the corresponding antigen receptors, or vice versa, needs to be elucidated.

Additional striking features of SARS-CoV-2 are encouraged to be discussed in the light of APOBECs pressure. The genome of SARS-CoV-2 seems to avoid cytosine, thus being depleted of this nucleotide (25). In the article by Danchin A and Marliere P (25), the emphasis is given to the unique role of cytosine-related metabolic processes in coordination with the antiviral response against RNA viruses; the loss of cytosine nucleotides in viral genomes, in particular within CpG motifs, may modulate the action of cellular factors such as viperin (virus inhibitory protein, endoplasmic reticulum-associated, IFN-inducible), of ZAP (the host zinc-finger antiviral protein), or of Dnmt2 (a DNA methyltransferase). Viewing from another angle, one could set up the hypothesis that SARS-CoV-2-reduced cytosine content is an escape strategy counteracting the efficient APOBEC-driven antiviral actions.

The APOBEC-driven C-to-U substitution might furthermore be a decisive factor for disease severity. In fact, the increase in U in the viral genome potentiates the activation of TLR7 and TLR8, which in turn trigger the production of pro-inflammatory cytokines such as TNF-α and IL-6 (21). Worth discussion is the consequence of such U-rich SARS-CoV-2 variants. When kept under control, an activation of pattern recognition receptors might be beneficial for efficient antiviral immune response. However, a hyperstimulation may trigger a cytokine storm leading to severe and life threatening COVID-19.

## AID as Master Regulator of Germinal Center Reactions and Genome-Wide Mutator

Within the herein discussed AID/APOBEC family, an outstanding role is attributed to the molecule AID (encoded by *AICDA* gene) discovered by Tasuku Honjo’s group (26, 27). From our point of view, AID can be seen as the prototype of a cellular master regulator. This rather small molecule, a 198 amino acid protein, is determinative for the power of the humoral arm of the adaptive immune response (26). Central to its decisive role in B-cell biology is the ability of AID to target immunoglobulin genes and drive two complementary processes for protective immunity – the somatic hypermutation and the class-switch recombination. Both DNA editing events share a common mechanism with the AID-driven deamination of single-stranded DNA and both are essential for a powerful antibody-driven immune response. In the course of infection, somatic hypermutation, also known as affinity maturation, is the process responsible for the production of high-affinity antibodies, which are, due to the stronger and more stable binding to the corresponding antigen, more potent in their mode of action including the neutralizing potential. Class-switch recombination events are linked to the production of immunoglobulins of various isotypes, which differ in their Fc regions and thus are the drivers of a multitude of diverse immune responses (28). Of increasing interest is the location of action of AID-expressing B cells – the above described events take place within highly organized and specialized structures known as Germinal Centers (GCs) of lymphoid structures. The strictly coordinated sequence of events, which includes active cellular participants such as mature dendritic cells, T cells, follicular helper T cells, follicular dendritic cells, and, as central actors, the B cells, ensures the desired outcome of a GC reaction – the production of high affinity antibodies of various isotypes by plasma cells or memory B cells (29, 30).

A recent study by Kaneko N et al. (31) demonstrates the link between loss of GCs in lymph nodes and COVID-19. Besides the secondary lymphoid organs such as lymph nodes and spleen, the lymphoid structures are crucial players of the immune response at the mucosal sites such as the gut-associated lymphoid tissue (GALT) of the digestive tract or the bronchus-associated lymphoid tissue (BALT) in the lung (32). Intriguingly, both locations are well known target sites for SARS-CoV-2 (33, 34). In this respect, we hypothesize that the person-specific characteristics of lymphoid structures, which among others include the AID expression and activity, might be linked to COVID-19 progression and severity. In analogy, this holds true for isolated lymphoid structures in the colon mucosa and the disease outcome for patients with metastatic colorectal cancer (35). We therefore propose that, if converted to measurable numerical values, the patient-specific immune phenotype of lymphoid structures could be used as a novel patient stratification strategy. Additively, the AID-positive GCs were shown to be formed locally in nasal polyp tissues and were able to produce on-site antibodies in patients with chronic rhinosinusitis with nasal polyps (36). In the context of the theme of the article, the question arises whether there is an association between the presence of nasal polyps, local formation of lymphoid structures with active AID, and the tissue/location prevalence for SARS-CoV-2 infection and/or the disease severity.

A further hallmark to emphasize is the ability of AID to target non-immunoglobulin genes in non-B cells. In circumstances such as chronic inflammation or cancer, AID was shown to act as a genome-wide mutator, causing genetic alterations in cancer-related genes (37–39). Some viruses have been shown to induce AID expression in both B cells and cells of non-B-cell origin (40–42). In its turn, there is evidence that AID may impact viral fitness (43, 44).

## Mapping the AID/APOBEC Gene Expression Signature in a Patient-Specific Manner

**Figure 2:**
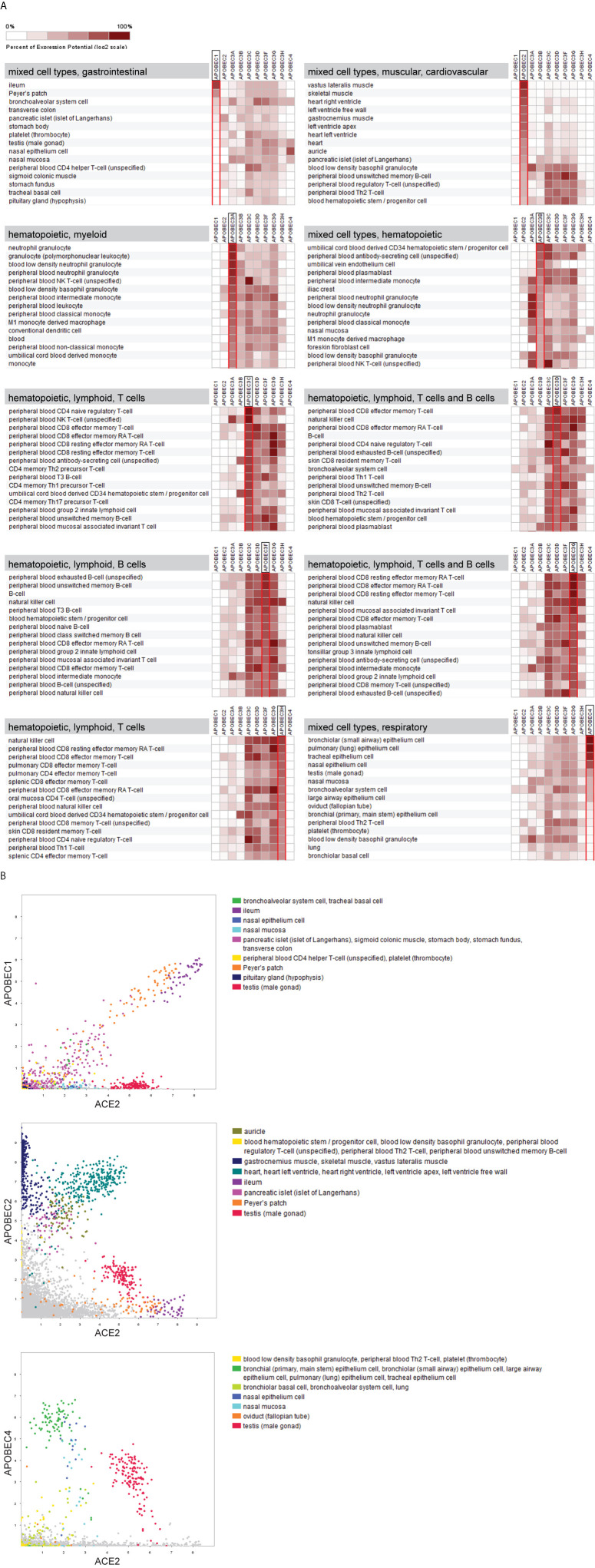
The tissue- and cell type-specific gene expression signature of APOBECs. Comprehensive analysis of mRNASeq data sets was performed across 208 anatomical parts under healthy conditions covering various tissues and cell types (in total 8.856 data sets). **(A)** Heat maps illustrate the gene expression levels of the 10 *APOBECs* in their respective 15 highest expressing tissues and cell types. Results are sorted according to the expression level of the indicated *APOBEC* family member. The affiliation of the predominant cell types/tissues is indicated as heading in the grey box. Data was extracted from GENEVESTIGATOR on March 24, 2021. **(B)** 2-Gene plots visualize the interrelated expression of *ACE2* (x-axis) and either *APOBEC1* or *APOBEC2* or *APOBEC4* (all y-axis) across the above indicated data selection. The 15 highest expressing tissues and cell types for the corresponding *APOBEC* family member (shown in A) are highlighted by different colors. GENEVESTIGATOR-based analysis was performed on March 29, 2021.

The AID/APOBECs possess a highly conserved zinc-dependent deaminase domain but are extremely diverse in function. Members of the APOBEC family show different tissue- and cell type-specific gene expression patterns (**Figure 2**). This does not mean, however, that one particular cell type expresses only one particular APOBEC, but rather that each cell type has its characteristic APOBEC repertoire.

Intriguingly, our comprehensive analysis of transcriptomic data sets revealed that *APOBEC* family members respectively show preferential expression in a particular cell or tissue type (**Figure 2A**). More specifically, the *APOBEC*-based expression pattern *per se* enables the differentiation between myeloid (*APOBEC3A*) and lymphoid (*APOBEC3C*/*D*/*F*/*G*/*H*) lineages and, within the latter, between B cells and T cells.

For some members, the expression pattern aligns with the proven functional pattern. For example, *APOBEC2* shows preferential expression in skeletal muscle and heart and has been shown to play roles in skeletal and cardiac muscle differentiation [(45–47) and our data from a compendium-wide analysis shown in **Figure 2A**]. These observations suggest that the bodily expression pattern of APOBECs might be helpful for predicting their *in vivo* function.

A prime example among APOBECs is APOBEC4 – of which the expression map and function are both poorly characterized. *APOBEC4* expression in mouse testis was shown by Rogozin et al. (48) and by the human data presented in this study (**Figure 2A**) and is suggestive for its role in spermatogenesis. By analyzing large transcriptomic data sets, covering the vast majority of cell types and tissues available from shared scientific resources, we strikingly found high expression of *APOBEC4* in cell types and anatomical parts targeted by SARS-CoV-2. This includes bronchiolar epithelial cells, pulmonary epithelium cells, tracheal epithelium cells, and nasal epithelium cells, all ranked among the highest expressing cell types for *APOBEC4* (**Figure 2A**). This raises the question about its potential associations with disease-affected organs and/or disease severity.

For *APOBEC1*, *APOBEC2*, and *APOBEC4*, the family members which show preferential expression in non-hematopoietic cells, we aligned their gene expression patterns with the one of angiotensin-converting enzyme 2 (*ACE2*), encoding the entry receptor for SARS-CoV-2 (49, 50). Of particular interest are those tissues and/or cell types which are characterized by high expression levels of both molecules – the *APOBEC* member and *ACE2* (**Figure 2B**). These include the gastrointestinal tract, primarily the ileum, for *APOBEC1*; the heart and the testis for *APOBEC2*; the epithelial cells of the respiratory system and the nasal epithelium as well as the testis for *APOBEC4*. These novel findings further interrelate the APOBECs and SARS-CoV-2.

An ambitious but clinically relevant scenario would be to characterize the patient- or patient group-specific antiviral cell state attributed to the AID/APOBECs. This could cover their expression patterns in various cell types of different origin, assigned to distinct tissues, thereby depicting the cell type-specific *AID*/*APOBEC* gene signature. Considering the protein homology issue among APOBECs and therefore the challenge of mapping their expression using antibodies directed to individual family members, the use of public transcriptomic data sets could provide corresponding insights. For example, platforms such as GENEVESTIGATOR (https://genevestigator.com/) curate and consolidate publically available studies from microarrays, mRNA sequencing and single cell transcriptomics for a fine granular representation of a multitude of cell types and tissues under normal, healthy conditions versus perturbations and diseased states. Previously, we used this strategy for the comprehensive analysis of the role of AID/APOBECs in the pathobiology of complex multifactorial diseases, including immune-/inflammatory-based and cancer (6, 36, 51). The wide-range applicability of the developed integrative strategy for various multifactorial diseases was highlighted in the book chapter entitled “An Integrative MuSiCO Algorithm: From the Patient-Specific Transcriptional Profiles to Novel Checkpoints in Disease Pathobiology” (52).

## Mapping the AID/APOBEC Polymorphisms in a Patient-Specific Manner

In addition to the patient-orientated *AID*/*APOBEC* gene expression signature, the polymorphism analysis of individual family members should be strongly considered. In fact, variations have been detected on the population level. Several studies indicate that polymorphisms in APOBEC3 subfamily members – APOBEC3D, APOBEC3F, APOBEC3G and, in particular, APOBEC3H – likely impact HIV-1 replication, what in turn may be correlative with infection risk and disease progression as shown for South African HIV-1-infected cohort (53). APOBEC3H is among the most genetically diverse across the APOBEC3 subfamily and includes seven haplotypes, some of which have shown individual functional characteristics in HIV-1-infected cells (54). Important to emphasize is the existence of bidirectional interrelations between host endogenous APOBEC3s and the viral system, represented for HIV-1 by the Vif protein, a potent regulator of viral infectivity. This mechanism is likely among adapting mechanisms to reach host-virus equilibrium. Although SARS-CoV-2 does not have Vif protein analogs, the potential impact of APOBEC3 haplotypes to individual differences in infection risk or COVID-19 severity has been addressed recently (55).

Additionally, we would like to bring attention to the 29.5-kb common human deletion polymorphism that occurs between *APOBEC3B* and *APOBEC3A*. The deletion was discovered by two independent approaches (56, 57) and a detailed sequence analysis was performed by population genetic analysis across continental groups (51 populations; 1,277 DNA samples) (58). The resulting effect is characterized by a loss of *APOBEC3B* and potential alterations in regulation of *APOBEC3A*. By mapping the deletion profiles to different geographies, the authors could illustrate drastic differences in the frequency of the deletion around the world with significantly elevated values (frequencies ranging from 0.9% to 92.9%) when moving eastward from Africa. Thus, the deletion was found to be rare in Africans and Europeans, more common in East Asians and Amerindians, and almost fixed in Oceanic populations. The reason(s) why APOBEC3A/B deletion polymorphism is stratified in the human population is not yet clear as well as the functional consequences on antiviral response. Given the very special role of APOBEC3 subfamily members as intrinsic antiviral factors, the association of removal of APOBEC3B and of modulated APOBEC3A with the pathobiology of COVID-19 remains to be elucidated.

## Concluding Remarks

In this perspective – on the basis of systematic literature search and own novel findings – we addressed key aspects interrelating the AID/APOBECs and SARS-CoV-2. The presence of multilevel complexity underlying COVID-19 as multifactorial disease, with multiple interrelated pathomechanisms acting in parallel, multiplied by the complexity of the AID/APOBEC family, which is characterized by cell/tissue type-specific expression patterns, polymorphisms, DNA/RNA targeting specificity, and a plethora of antiviral functions, calls for the necessity of implementation of a holistic approach. As part of personalized/precision medicine, such a systems biology-based strategy is essential for our understanding of the patient-specific nature of the AID/APOBEC imprint linked to disease pathobiology with a perspective for the translational applicability of the knowledge.

## Data Availability Statement

The original contributions presented in the study are included in the article/supplementary material, further inquiries can be directed to the corresponding authors.

## Author Contributions

AM: conceptualization, writing‐original draft, data mining, and preparing figures. PP: conceptualization and writing‐original draft. PZ: writing‐original draft and data mining. IR: conceptualization and writing‐original draft. DM: conceptualization and writing‐original draft. All authors contributed to the article and approved the submitted version.

## Funding

This work was supported by the Medical and Scientific Fund of the Mayor of the Federal Capital Vienna (Medizinisch-Wissenschaftlicher Fond des Bürgermeisters der Bundeshauptstadt Wien) / the Foundation Fund to Promote the Fight against Tuberculosis and other Lung Diseases, Vienna, Austria (Stiftungsfond zur Förderung der Bekämpfung der Tuberkulose und anderer Lungenkrankheiten), project Nr. COVID043. IR was supported by the Intramural Research Program of the National Library of Medicine at the National Institutes of Health.

## Conflict of Interest

Author PZ was employed by Nebion AG.

The remaining authors declare that the research was conducted in the absence of any commercial or financial relationships that could be construed as a potential conflict of interest.
